# Effects of Carrier Trapping and Noise in Triangular-Shaped GaN Nanowire Wrap-Gate Transistor

**DOI:** 10.3390/nano15171336

**Published:** 2025-08-30

**Authors:** Siva Pratap Reddy Mallem, Peddathimula Puneetha, Yeojin Choi, Mikiyas Mekete Mesheha, Manal Zafer, Kab-Seok Kang, Dong-Yeon Lee, Jaesool Shim, Ki-Sik Im, Sung Jin An

**Affiliations:** 1 Advanced Material Research Center, Kumoh National Institute of Technology, Gumi 39177, Republic of Korea; drmspreddy@kumoh.ac.kr; 2 Department of Robotics and Intelligent Machine Engineering, College of Mechanical and IT Engineering, Yeungnam University, Gyeongsan 38541, Republic of Korea; puneethaphd@gmail.com (P.P.); dylee@ynu.ac.kr (D.-Y.L.); 3 Department of Materials Science and Engineering, Kumoh National Institute of Technology, Gumi 39177, Republic of Korea; dota23@kumoh.ac.kr (Y.C.); mikimekete2@gmail.com (M.M.M.); manalzafer2@gmail.com (M.Z.); 4 Department of Electrical Engineering, Daegu Campus, Korea Polytechnics, Daegu 41765, Republic of Korea; seok0826@kopo.ac.kr; 5 School of Mechanical Engineering, Yeungnam University, Gyeongsan 38541, Republic of Korea; 6 Department of Green Semiconductor System, Daegu Campus, Korea Polytechnics, Daegu 41765, Republic of Korea

**Keywords:** wrap-gate transistor, nanowire, GaN, core and surface, trap effects

## Abstract

The most widely used nanowire channel architecture for creating state-of-the-art high-performance transistors is the nanowire wrap-gate transistor, which offers low power consumption, high carrier mobility, large electrostatic control, and high-speed switching. The frequency-dependent capacitance and conductance measurements of triangular-shaped GaN nanowire wrap-gate transistors are measured in the frequency range of 1 kHz–1 MHz at room temperature to investigate carrier trapping effects in the core and at the surface. The performance of such a low-dimensional device is greatly influenced by its surface traps. With increasing applied frequency, the calculated trap density promptly decreases, from 1.01 × 10^13^ cm^−2^ eV^−1^ at 1 kHz to 8.56 × 10^12^ cm^−2^eV^−1^ at 1 MHz, respectively. The 1/*f*-noise features show that the noise spectral density rises with applied gate bias and shows 1/*f*-noise behavior in the accumulation regime. The fabricated device is controlled by 1/*f*-noise at lower frequencies and 1/*f*^2^-noise at frequencies greater than ~ 0.2 kHz in the surface depletion regime. Further generation–recombination (G-R) is responsible for the 1/*f*^2^-noise characteristics. This process is primarily brought on by electron trapping and detrapping via deep traps situated on the nanowire’s surface depletion regime. When the device works in the deep-subthreshold regime, the cut-off frequency for the 1/*f*^2^-noise characteristics further drops to a lower frequency of 30 Hz–10^4^ Hz.

## 1. Introduction

Planar metal-oxide semiconductor field-effect transistors (MOSFETs), based on silicon (Si), have dominated the global semiconductor market since the first integrated circuit (IC) was invented in 1958 [1,2]. They are still used to construct microelectronic devices like contemporary microprocessors, which can integrate over one billion transistors on a single IC [3,4]. Alternative device architectures are necessary to attain better integration level and reduce power consumption in ICs as MOSFETs scaling approaches the end of Moore’s law roadmap [1,2,3,4]. Devices that function as switches and regulate the flow of electricity are typically needed for power electronics. To reduce off-state leakage, a power switching device should ideally have high breakdown voltages and very little current flow in the off-state (*I*_off_) [5]. To reduce conduction losses, the device should be able to have a high maximum current while maintaining low on-resistance (*R*_on_) [5,6]. Over the past few decades, Si has been the preferred semiconductor material for power devices because of its abundant availability and ease of manufacturing [5,7]. However, alternative semiconductor materials are required since Si is theoretically limited as a channel material [8]

For power amplifier applications, wide-bandgap materials like gallium nitride (GaN) offer exceptional characteristics like high power operation, high frequency, and high conductivity [9,10,11,12]. High-electron-mobility transistors (HEMTs) of the AlGaN/GaN combination can function well at high breakdown voltage in the kV range [13]. The gate–drain spacing must be increased, which will result in a larger chip and greater fabrication costs, to attain a high breakdown voltage in the planar device [14]. Furthermore, surface trapping and substrate leakage limit the performance of planar devices. 

Recently, nanowire FET research has expanded in response to these practical challenges [15]. Nanowire FETs are good options for next-generation nano-electronics/optoelectronics applications due to their distinct physical/chemical characteristics [16,17]. Researchers from all around the world are drawn to the nanowire wrap-gate transistor among the new alternative’s topologies [18,19]. This is because of its high integrating density, excellent electrostatic control over the channel, and reduced short channel effects [20]. These days, GaN nanowire wrap-gate transistors are developed because of their high surface-to-volume ratio and enhanced electrostatic control on channel [21]. In addition, these triangular-shaped nanowire transistors have the minimum cross-sectional area under the similar line width case and hence have foremost electrostatic dominion capability and can acquire significant device performance compared to square- and circular-shaped nanowire transistors [22].

In fact, nanowire devices’ electrical properties are typically restricted by carrier traps or defects that originate at the nanowire’s core/surfaces. As generation–recombination (G–R) centers, carrier trap levels or deep trap levels start close to the mid-gap energy and could affect carrier transit [23,24]. Trapped charges induce fluctuations in the carrier mobility, electric field, diffusion coefficient, space charge region width, barrier height, etc. Therefore, to control the electrical properties of nanowire devices, it is critical to understand trap behavior. Understanding the effects of trapping features at the surface and/or in the core of the double-transferred GaN nanowire channels on the devices’ performance in this study requires an understanding of the capacitance and conductance values based on frequency, along with the related noise characteristics. The main objective of this study is to use capacitance conductance characteristics seen in the frequency range of 1kHz to 1MHz to investigate the carrier trap characteristics at the nanowire’s surface/core. The second objective is to estimate the surface trap density. Finally, the 1/*f*-noise properties of the one-dimensional GaN nanowire wrap-gate transistor are evaluated.

## 2. Materials and Methods

**Figure 1:**
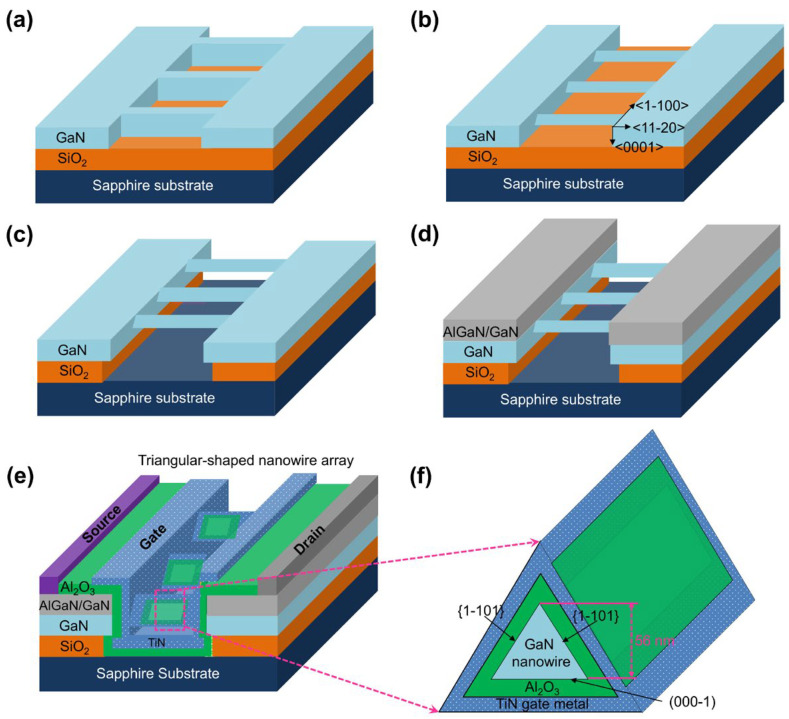
Fabrication process for triangular-shaped GaN nanowire wrap-gate transistor: (**a**) the separation of GaN nanowires; (**b**) reduction of GaN nanowire arrays by chemical etchant along $\left\{1\bar{1}01\right\}$ direction, resulting in triangular-shaped nanowire; (**c**) separation of triangular-shaped GaN nanowire arrays by etching the SiO2 layer; (**d**) deposition of AlGaN/GaN layer on the patterned surface; (**e**) schematic illustration of triangular-shaped GaN transistor; (**f**) a cross-section diagram of the triangular-shaped GaN nanowire.

Soitec’s Smart Cut^TM^ technology was used to fabricate the GaN on insulator (GaNOI) structure using dual-wafer-transfer procedures [24]. A GaN film layer (150 nm) and oxide layer (800 nm) were positioned on a sapphire (Al_2_O_3_) substrate to form the 4-inch GaNOI wafer structure. The triangular-shaped GaN nanowire transistor fabrication procedure was as follows (Figure 1). Using polymethyl methacrylate (PMMA) solution on the surface of the GaNOI wafer structure, electron-beam (E-beam) lithography was employed to form a pattern along the $\langle 11\bar{2}0\rangle$ direction to define the initial active region (Figure 1a). TMAH solution was applied to the patterned GaN stripe for 10 min at 90 °C to avoid plasma damage and minimize surface roughness. Due to the strong anisotropic chemical nature of the TMAH solution, it etches in the lateral direction without causing any vertical etching direction $\langle 0001\rangle$ (Figure 1b). A triangular-shaped one-dimensional GaN nanowire with a height of approximately 56 nm and sloped sidewall surfaces corresponding to $\langle 1\bar{1}00\rangle \;$occurred by using the tetramethylammonium hydroxide (TMAH) solution along the $\langle 1\bar{1}01\rangle \;$direction, which decreased the stripe width. To fully remove the oxide beneath the one-dimensional GaN nanowire, the wafer must be dipped in a buffered oxide etch (BOE) solution (Figure 1c). 

MOCVD was used to deposit an undoped GaN film (50 nm) and an Al_0.3_Ga_0.7_N film (20 nm) in certain regions on the patterned GaN wafer surface (Figure 1d). The *r*-plane on the patterned GaN wafer surface experiences a self-limiting growth process along its $\left\{1\bar{1}01\right\}\;$direction. N atoms on the surfaces of the *r*-plane simply interacted with H atoms to create N–H bonds during the MOCVD growth process, stabilizing the plane and limiting the growth. Because of the $\left\{1\bar{1}01\right\}\;$crystal direction, the AlGaN/GaN films were therefore only successfully deposited in the source/drain (S/D) regimes and not on the surface of GaN nanowires; as a result, the one-dimensional GaN nanowire’s area remained unchanged. Regrowing AlGaN/GaN films in the source/drain area has the benefit of depositing a two-dimensional electron gas (2DEG), which lowers the device’s series resistance.

Next, utilizing plasma-enhanced chemical vapor deposition (PECVD) technology, Al_2_O_3_ (20 nm) and TiN (10 nm) films were chosen as an insulating gate material and for the metal-gate electrode, respectively, and deposited sequentially. Ti/Al/Ni/Au metal layers were then deposited using the E-beam deposition method after contact holes were unlatched to connect the S/D regions. The resultant metal layers were annealed using a rapid thermal process (RTP) system for 30 s in N_2_-gas flow. A high-resolution transmission electron microscope (HRTEM, JEOL, JEM-2100F, Tokyo, Japan) was used to analyze the device cross-section. Using a semiconductor source meter unit (B1500A, Agilent, Santa Clara, CA, USA), the current, capacitance, and conductance values of the one-dimensional GaN nanowire wrap-gate transistor were measured. Here, the B1500A semiconductor parameter analyzer can be used to characterize capacitance and conductance by employing its impedance measurement unit (IMU). The IMU applies a small AC signal at various frequencies to the device under test (DUT) and measures the resulting current. By analyzing the amplitude and phase relationship between the voltage and current, the B1500A can determine the capacitance and conductance of the DUT at each frequency. A completely automated noise measurement system (NOISYS7, Synergie Concept/Instrumentation & Electronic, Meylan, France) was used to measure the 1/*f*-noise.

## 3. Results

**Figure 2:**
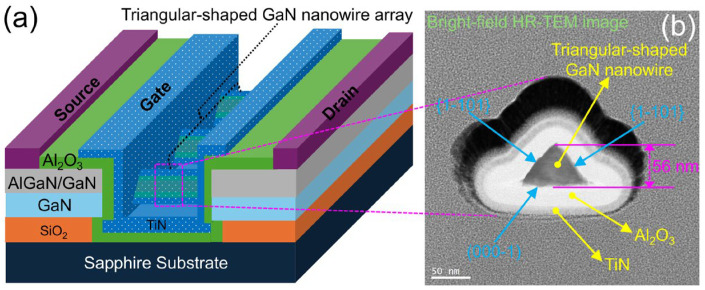
(**a**) The triangular-shaped GaN nanowire wrap-gate transistor’s schematic diagram. (**b**) A bright-field high-resolution transmission electron microscopy cross-sectional picture.

**Figure 3:**
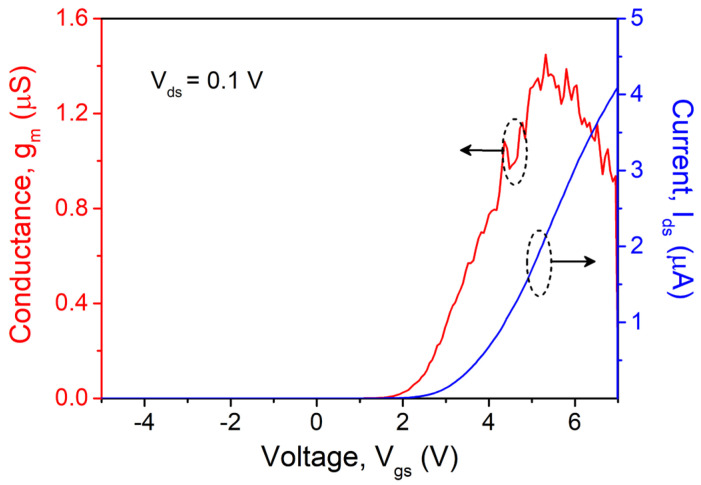
Linear data plots of the drain current (I_ds_) (i.e., right-side) and transconductance (g_m_) (i.e., left-side) vs. gate–source voltage (V_gs_) at applied drain voltage of V_ds_ = 0.1 V.

**Figure 4:**
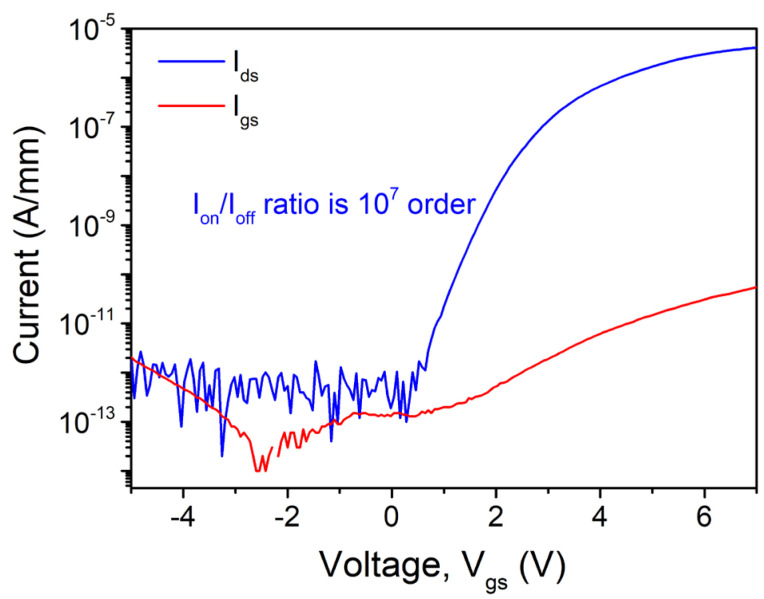
Data plots of I_ds_ and I_gs_ vs. V_gs_ at applied drain voltage of V_ds_ = 0.1 V.

The schematic device structure of the studied GaN nanowire wrap-gate transistor is shown in Figure 2a, which contains 64 one-dimensional GaN nanowires in the shape of triangles, each having two identical crystal faces $\left\{1\bar{1}01\right\}.$ A bright-field HR-TEM image on the right side of Figure 2b makes it evident that the AlGaN/GaN nanowire core is triangular-shaped and surround by gate oxides (Al_2_O_3_) and gate metal (TiN). The triangular-shaped wrap-gate GaN nanowire transistor normally operates off with a threshold voltage (*V*_th_) of approximately 3.5 V, as seen in Figure 3. This high value is potentially achieved because of the fully depleted triangular-shaped wrap-gate GaN nanowire. At *V*_gs_ = 7 V and *V*_ds_ = 0.1 V, the highest transconductance (*g*_m_) peak is around 0.94 μS, while the maximum drain current (*I*_ds_,_max_) is approximately 4.1 μA. Additionally, Figure 4 shows the semi-logarithmic transfer characteristics. The leakage current of the triangular-shaped wrap-gate GaN nanowire transistor is as low as around 10^−13^ A/mm. The *I*_on_/*I*_off_ ratio of 10^7^ is high for the triangular-shaped wrap-gate GaN nanowire transistor. Further, the field-effect carrier mobility of the double-transferred wrap-gate GaN nanowire transistor is 15 cm^2^/V·s. Furthermore, the subthreshold swing of the fabricated device is 130 mV/decade. 

**Figure 5:**
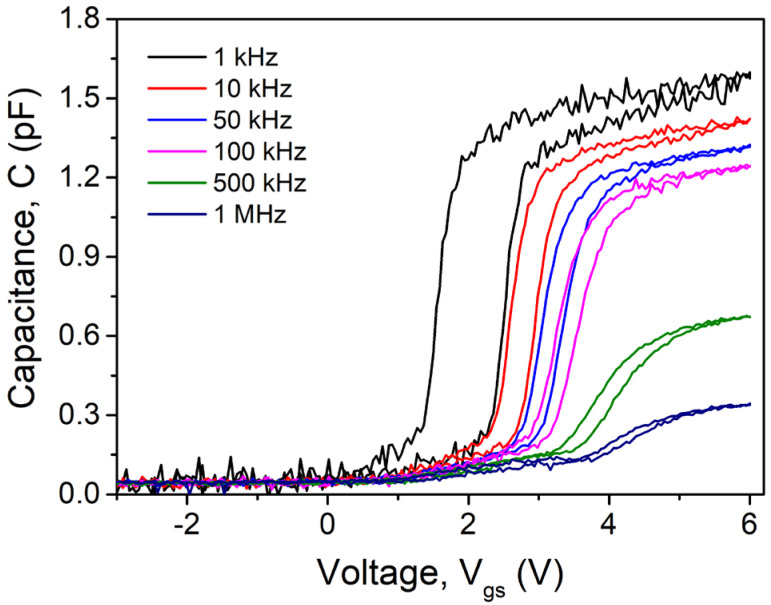
Frequency-dependent capacitance (C) vs. gate–source voltage (V_gs_) of the measured transistor.

Three distinct voltage-dependent regimes are seen in the frequency-dependent capacitance characteristics of the triangular-shaped wrap-gate GaN nanowire transistor measured at the comparatively low frequency of 1 kHz, as shown in Figure 5: accumulation, surface depleted and fully depleted regimes of the whole GaN nanowire channel. Because of the very long lifetime in the case of GaN-related materials, the surface inversion at *V*_gs_ < 0 V has been eliminated [24,25]. As the frequency increases, it is observed that the triangular-shaped wrap-gate GaN nanowire transistor shows a significant positive shift in flat-band voltage (*V*_FB_) with grievous frequency dispersion. There is also a distinguishable hysteresis of the shift in *V*_FB_ (i.e., Δ*V*_FB_) between the bias sweeps, but the Δ*V*_FB_ falls with rising frequency. The existence of many surface traps with different lifetimes is reflected in this extreme frequency dispersion. A substantial interface trap capacitance (*C*_it_) is associated with this; the traps respond effectively to low and intermediate frequencies of alternating current (AC) signals, but they barely react at high frequencies [26,27]. The frequency scattering may also be brought about by the deep-level traps that were added to the GaN nanowire during the previously mentioned dual-wafer-transfer process. A severe dispersion in the accumulation regime and a positive shift in *V*_FB_ with increasing frequency result from the trapped carriers’ inability to respond to the AC signal at high frequencies, which prevents them from contributing to the current movement and drastically lowers the determined capacitance [24,28].

**Figure 6:**
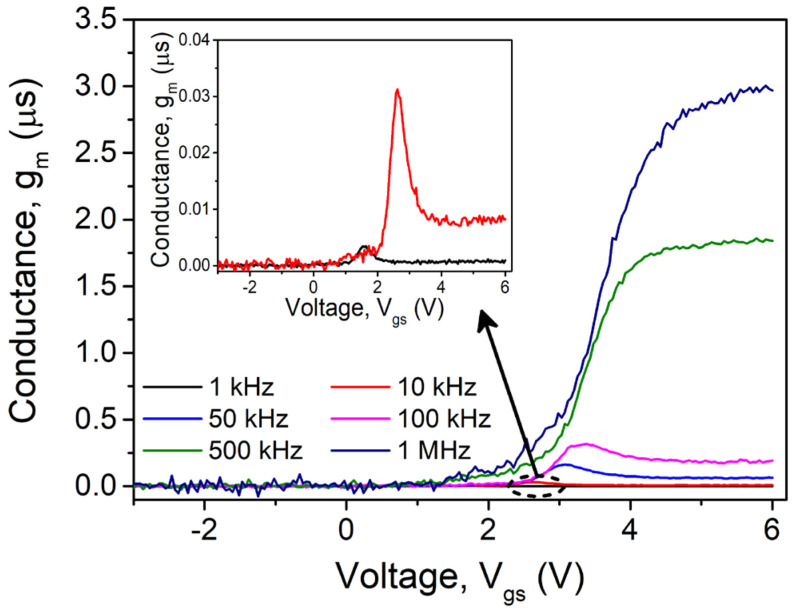
Frequency-dependent conductance (*g*_m_) vs. gate–source voltage (*V*_gs_) of measured transistor.

The frequency-dependent conductance characteristics are displayed in Figure 6. In opposition to the capacitance properties, the conductance plots shift to negative bias, and the conductance rises with rising frequency. Additionally, it is observed that a conductance peak develops at low frequencies (less than 100 kHz) but vanishes at higher frequencies (500 kHz and 1 MHz) when *V*_gs_ < *V*_th_. The presence of surface traps with different time responses, as mentioned in the capacitance measurement, is the exact same source of conductance behavior. This indicates that the traps may readily follow the AC wave signal at low frequencies (the value of the period is greater than the carrier lifetime of charge in the traps), which subsequently clearly contributes to the conductance of the device [29,30]. In comparison with low frequencies (> 500 kHz), the traps are nearly impossible to follow at high frequencies and do not contribute to the observed conductance. The charges in surface-level traps cannot ensure the AC wave signal at prominent frequencies because the time constant is significantly greater than the period, unlike at low frequencies. 

**Figure 7:**
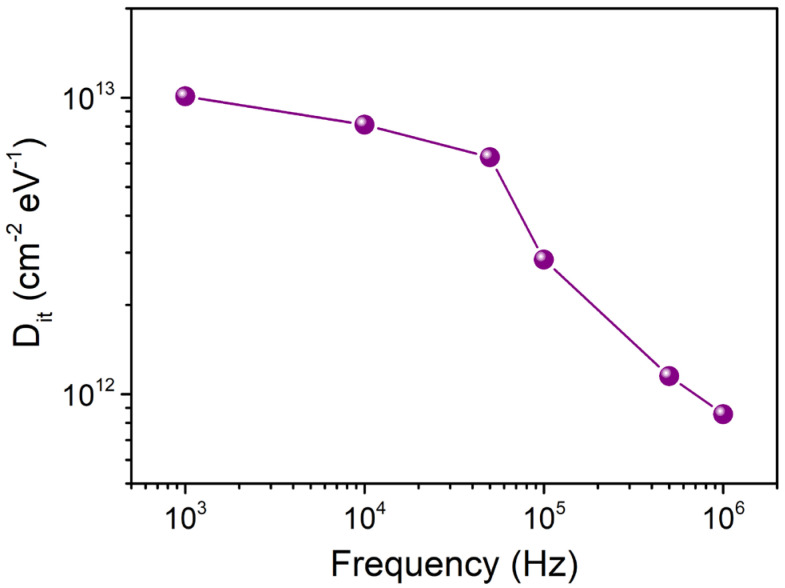
Plot of frequency-dependent interface trap density (D_it_).

Using capacitance and conductance values, the Hill–Coleman technique is used to assess the frequency-dependent interface trap density (D_it_) values [31]. The following technique can be used to determine the D_it_ values:

(1) $$D_{it}=\frac{2}{qA}\left(\frac{{\left(g_{m}/\omega \right)}_{max}}{{\left({\left(g_{m}/\omega \right)}_{max}C_{OX}\right)}^{2}+{\left(1-C/C_{OX}\right)}^{2}}\right)$$

where q, A, and *C*_OX_ are charge, area of contact, and oxide capacitance (i.e., determined from capacitance/conductance measurements in strong accumulation regime at maximum applied frequency range (1 MHz)). As Figure 7 illustrates, the extracted *D*_it_ values gradually drop with increasing frequency. Additionally, it is seen that the *D*_it_ values derived from Equation (1) are comparable to those derived from the typical low-frequency oxide/nitride-semiconductor contacts. The significant dispersion effects on the capacitance and conductance properties are caused by the high value of *D*_it_. Here, *D*_it_ deactivation with increasing frequency can account for the rise in *V*_FB_ and fall in Δ*V*_FB_ with rising frequency, as illustrated in Figure 5 and Figure 6.

**Figure 8:**
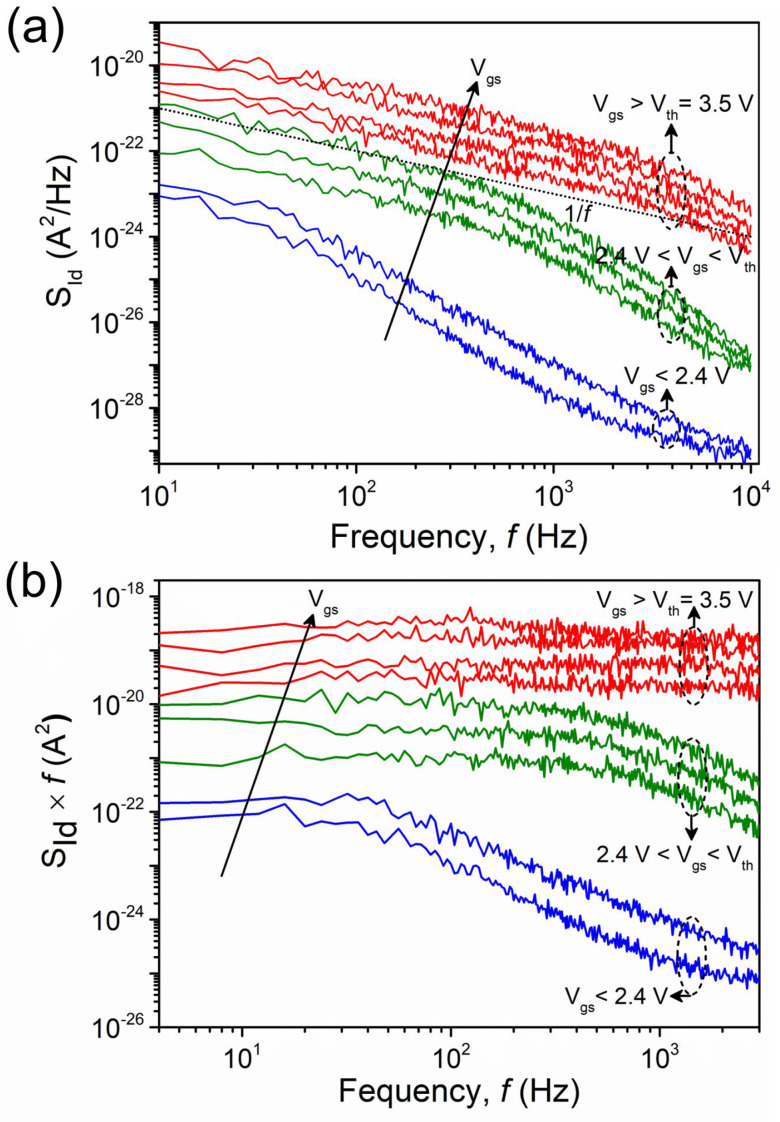
(**a**) Noise spectral density (S_Id_) and (**b**) *S*_Id_ × *f* values according to the frequency of the fabricated transistor at *V*_ds_= 0 1 V.

Next, measurements of low-frequency noise are taken to better examine the trapping effect in the triangular-shaped wrap-gate GaN nanowire transistor. It is commonly known that 1/*f*-noise measurements can give accurate data on the nature of carrier conduction in bulk materials and at the surface when combined with other electrical characteristics. The 1/*f*-noise measurements are employed to assess the quality of semiconductor devices and investigate impurity and deep-level traps in crystalline semiconductors [32,33]. In the frequency range of 4–10^4^ Hz at *V*_ds_ = 0.1 V, the noise spectral density (*S*_Id_) of the voltage deviations varied regarding frequency. The bias varied from the deep-subthreshold regime to strong accumulation. The *S*_Id_ increases with gate voltage, as seen in Figure 8a. As previously mentioned in the capacitance and conductance measurements, the noise is primarily caused by the carrier transport between the shallow states and the accumulation channel; as evidenced by the noise curves, there is an obvious 1/*f* shape in all measured frequency regimes in the extreme accumulation regime (*V*_gs_ > *V*_th_ = 3.5 V).

However, the noise spectra in the depletion regime (2.4 V < V_gs_ < V_th_) take on a 1/*f* shape at lower frequencies and change to a 1/*f*^2^ shape at frequencies greater than about 0.2 kHz. The G–R noise resulting from the carrier transport mechanism via deep-level traps, which have a longer time constant but a comparatively shorter one than the surface traps, is responsible for these 1/*f*^2^ curves. This indicates that the carriers undergo both surface and surface depletion regime trapping and detrapping in the GaN nanowire channel. The cut-off frequency (*f_c_*) for the 1/*f*^2^ curves moves to lower frequencies of 30 Hz–10^4^ Hz in the deep-subthreshold regime (*V*_gs_ < 2.4 V). 

The graph for the *S*_Id_ multiplied by the frequency versus frequency in Figure 8b clearly shows the 1/*f*^2^ shape. From the cut-off frequency for the 1/*f*^2^ curves, we can extract the time constant (τ_i_) using the following equation:

(2) $$S_{Id}\times f=K_{f}+\sum_{i=0}^{N}\frac{A_{i}f}{1+{\left(\frac{f}{f_{c}}\right)}^{2}},\;\tau_{i}=\frac{1}{2\pi f_{c}}$$

where *K_f_* is the flicker noise coefficient of the 1/*f* noise and A_i_ is the Lorentzian plateau value of G–R noise component. The calculated τ_i_ are 3.2 ms and 0.5 ms for the deep-subthreshold regime (*V*_gs_ < 2.4 V) in the surface depletion regime (2.4 V < *V*_gs_ < *V*_th_), respectively. In fact, the fully depleted GaN nanowire channel is dominated by the G–R-related carrier transport mechanism, which includes comparatively longer time constants [34,35]. The deep-level traps’ influence on capacitance and conductance values is consistent with their role in the noise characteristics.

## 4. Conclusions

Frequency-dependent capacitance and conductance values were used to examine the trapping effects and noise properties of a triangular-shaped wrap-gate GaN nanowire transistor that was created using a dual-transfer and top-down method. It was discovered that the capacitance, conductance, and noise properties are significantly influenced by the high trap density at the triangular-shaped GaN nanowire channel’s surface and core. The results can be described as follows: (i) The carrier conduction between the accumulation surface channel and the shallow traps is efficient in the accumulation area (*V*_gs_ < *V*_th_ = 3.5 V), exhibiting 1/*f*-noise behavior with applied gate biases. (ii) The carrier transport mechanism via the deep-level traps in the triangular-shaped GaN nanowire channel’s depletion regime (2.4 V < *V*_gs_ < *V*_th_) becomes effective in the surface depletion regime, resulting in G–R noise with 1/*f*^2^ plot at frequencies higher than ~5 kHz rather than 1/*f* shape at lower frequencies. (iii) In the deep-subthreshold regime (*V*_gs_ < 2.4 V), the G–R-based carrier transport mechanism prevails via the deep-level traps in the fully and deeply depleted triangular-shaped GaN nanowire channel, resulting in a reduction in cut-off frequency for 1/*f*^2^ noise to a lower range of 30 Hz ~10^4^ Hz. The evolution of high-efficiency GaN-based nanowire transistor for upcoming nano-level electronic devices requires an understanding of trap characteristics which these results help to provide.

## Data Availability

The data are available on reasonable request from the corresponding author.
